# Bis(2,3-diamino­pyridinium) succinate trihydrate

**DOI:** 10.1107/S1600536809026439

**Published:** 2009-07-15

**Authors:** Hoong-Kun Fun, Kasthuri Balasubramani, Chin Sing Yeap

**Affiliations:** a X-ray Crystallography Unit, School of Physics, Universiti Sains Malaysia, 11800 USM, Penang, Malaysia

## Abstract

In the title salt, 2C_5_H_8_N_3_^+^·C_4_H_4_O_4_^2−^·3H_2_O, the asymmetric unit contains a protonated 2,3-diamino­pyridinium cation, half of a succinate dianion (disposed about a centre of inversion), and one and a half water mol­ecules. One of the water mol­ecules is disordered over two sites with occupancies of 0.670 (17) and 0.330 (17). The other water mol­ecule has an occupancy of 0.5 (from refinement). The pyridine N atom of the 2,3-diamino­pyridine mol­ecule is protonated. The protonated N atom and one of the 2-amino H atoms are hydrogen bonded to the succinate anion through a pair of N—H⋯O hydrogen bonds, forming an *R*_2_^2^(8) ring motif. In the crystal, mol­ecules are consolidated into a three-dimensional network by N—H⋯O and O—H⋯O inter­actions.

## Related literature

For substituted pyridines, see: Pozharski *et al.* (1997 ▶); Katritzky *et al.* (1996 ▶); Jeffrey & Saenger (1991 ▶); Jeffrey (1997 ▶); Scheiner (1997 ▶). For related structures, see: De Cires-Mejias *et al.* (2004 ▶); Fun & Balasubramani (2009 ▶); Balasubramani & Fun (2009*a* ▶,*b* ▶). For the stability of the temperature controller used in the data collection, see: Cosier & Glazer (1986 ▶). For hydrogen-bond motifs, see: Bernstein *et al.* (1995 ▶).
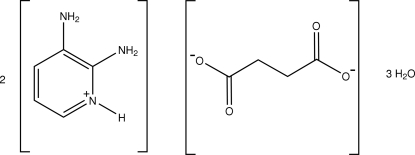


## Experimental

### 

#### Crystal data

2C_5_H_8_N_3_^+^·C_4_H_4_O_4_^2−^·3H_2_O
*M* *_r_* = 195.20
Monoclinic, 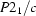

*a* = 12.7159 (4) Å
*b* = 3.9024 (1) Å
*c* = 18.7734 (6) Å
β = 94.933 (2)°
*V* = 928.13 (5) Å^3^
*Z* = 4
Mo *K*α radiation
μ = 0.11 mm^−1^
*T* = 100 K
0.17 × 0.13 × 0.06 mm

#### Data collection

Bruker SMART APEXII CCD area-detector diffractometer
Absorption correction: multi-scan (*SADABS*; Bruker, 2005 ▶) *T*_min_ = 0.981, *T*_max_ = 0.993
10934 measured reflections
2121 independent reflections
1364 reflections with *I* > 2σ(*I*)
*R*_int_ = 0.056

#### Refinement

*R*[*F* ^2^ > 2σ(*F* ^2^)] = 0.062
*wR*(*F* ^2^) = 0.129
*S* = 1.06
2121 reflections
178 parameters
H atoms treated by a mixture of independent and constrained refinement
Δρ_max_ = 0.26 e Å^−3^
Δρ_min_ = −0.24 e Å^−3^

### 

Data collection: *APEX2* (Bruker, 2005 ▶); cell refinement: *SAINT* (Bruker, 2005 ▶); data reduction: *SAINT*; program(s) used to solve structure: *SHELXTL* (Sheldrick, 2008 ▶); program(s) used to refine structure: *SHELXTL*; molecular graphics: *SHELXTL*; software used to prepare material for publication: *SHELXTL* and *PLATON* (Spek, 2009 ▶).

## Supplementary Material

Crystal structure: contains datablocks global, I. DOI: 10.1107/S1600536809026439/tk2489sup1.cif

Structure factors: contains datablocks I. DOI: 10.1107/S1600536809026439/tk2489Isup2.hkl

Additional supplementary materials:  crystallographic information; 3D view; checkCIF report

## Figures and Tables

**Table 1:** Hydrogen-bond geometry (Å, °)

*D*—H⋯*A*	*D*—H	H⋯*A*	*D*⋯*A*	*D*—H⋯*A*
N2—H2*N*2⋯O1^i^	0.90 (3)	2.14 (3)	2.978 (3)	155 (3)
N3—H1*N*3⋯O1^i^	0.85 (3)	2.14 (3)	2.993 (3)	176 (3)
N3—H2*N*3⋯O1*WA*^ii^	0.91 (3)	2.34 (3)	3.243 (6)	172 (2)
O1*WA*—H2*WA*⋯O2	0.85	1.93	2.764 (5)	165
N2—H1*N*2⋯O1	0.89 (3)	2.04 (3)	2.929 (3)	175 (2)
N1—H1*N*1⋯O2	0.96 (3)	1.69 (3)	2.643 (3)	171 (2)

Symmetry codes: (i) 
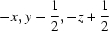
; (ii) 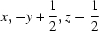.

**Fig. 1.**
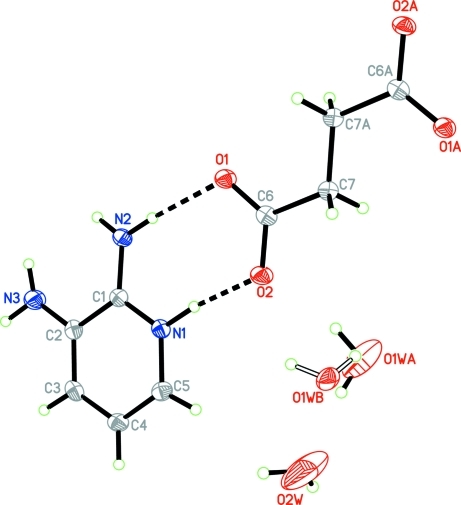
The molecular structure of (I), showing 50% probability displacement ellipsoids and the atom numbering scheme. Dashed lines indicate the hydrogen bonding. The O1 water molecule is disordered over two positions. Symmetry operation A:-x, 2-y, 1-z.

**Fig. 2.**
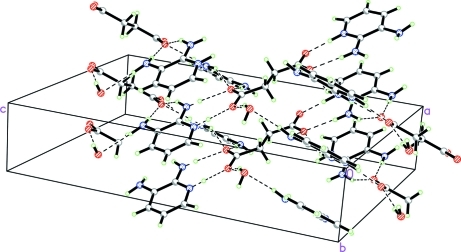
Part of the crystal packing showing the overall 3-D hydrogen-bonding network in (I). Dashed lines indicate the hydrogen bonding.

## supplementary crystallographic information

### Comment

Pyridine and its derivatives play an important role in heterocyclic chemistry (Pozharski *et al.*, 1997; Katritzky *et al.*, 1996). Further, pyridine and its substituted derivatives are often involved in hydrogen-bond interactions (Jeffrey & Saenger, 1991; Jeffrey, 1997;Scheiner, 1997). The crystal structures of 2,3-diaminopyridinium 4-hydroxybenzoate (Fun & Balasubramani, 2009), 2,3-diaminopyridinium 4-nitrobenzoate (Balasubramani & Fun, 2009*a*) and 2,3-diaminopyridinium benzoate (Balasubramani & Fun, 2009*b*) have been reported by us recently. In the hope to study some interesting hydrogen-bonding interactions, the title compound (I) was synthesized. Its molecular and crystal structure is presented here.

The asymmetric unit of (I) (Fig. 1), contains a protonated 2,3-diaminopyridinium cation, a half molecule of succinate anion (disposed about a centre of inversion), and one and half water molecules. In the 2,3-diaminopyridinium cation, protonatation N1 atom has lead to a slight increase (ca. 4 °) in the C1—N1—C5 angle to 123.6 (2)° compared with the unprotonated structure (De Cires-Mejias *et al.*, 2004). The 2,3-diaminopyridinium cation is planar, with a maximum deviation of 0.004 (2) Å for atom C2.

In the crystal packing (Fig. 2), the protonated N1 atom and a nitrogen atom of the 2-amino group (N2) are hydrogen-bonded to the succinate oxygen atoms (O2 and O1) *via* a pair of N—H···O hydrogen bonds forming a ring motif *R*_2_^2^(8) (Bernstein *et al.*, 1995). The 2-amino groups (N2 and N3) are involved in N—H···O hydrogen-bonding interactions to form a *R*_2_^1^(7) ring motif. The crystal structure is further stabilized by water molecules *via* O(water)—H···O and N—H···O(water) hydrogen bonding (Table 1 and Fig. 2).

### Experimental

An aqueous solution of hot methanol (10 ml/ 10 ml) of 2,3-diaminopyridine (27 mg, Aldrich) and succinic acid (29 mg, Merck) were mixed and warmed over a heating magnetic stirrer for 5 minutes. The resulting solution was allowed to cool slowly at room temperature. Crystals of (I) appeared from the mother liquor after a few days.

### Refinement

All the H atoms (other than the water H-atoms) were located from the difference Fourier map and allowed to refine freely [N–H = 0.85 (3)–0.96 (3) Å & C–H = 0.93 (2)–0.98 (2) Å]. The water H-atoms were located from the difference Fourier map but constrained to 0.85 Å from the parent atom with *U*_iso_(H) = 1.5*U*_eq_(O).

One water molecule has a refined occupancy of 0.495 (7) which was then fixed as 0.5 in the final refinement. The other water molecule is disordered (O1WA & O1WB) over two sites with occupancies of 0.670 (17) and 0.330 (17).

### Crystal data

**Table:**

2C_5_H_8_N_3_^+^·C_4_H_4_O_4_^2^^−^·3H_2_O	*F*(000) = 416
*M**_r_* = 195.20	*D*_x_ = 1.397 Mg m^−^^3^
Monoclinic, *P*2_1_/*c*	Mo *K*α radiation, λ = 0.71073 Å
Hall symbol: -P 2ybc	Cell parameters from 2108 reflections
*a* = 12.7159 (4) Å	θ = 2.2–30.0°
*b* = 3.9024 (1) Å	µ = 0.11 mm^−^^1^
*c* = 18.7734 (6) Å	*T* = 100 K
β = 94.933 (2)°	Block, brown
*V* = 928.13 (5) Å^3^	0.17 × 0.13 × 0.06 mm
*Z* = 4	

### Data collection

**Table:**

Bruker SMART APEXII CCD area-detector diffractometer	2121 independent reflections
Radiation source: fine-focus sealed tube	1364 reflections with *I* > 2σ(*I*)
graphite	*R*_int_ = 0.056
φ and ω scans	θ_max_ = 27.5°, θ_min_ = 2.6°
Absorption correction: multi-scan (*SADABS*; Bruker, 2005)	*h* = −16→16
*T*_min_ = 0.981, *T*_max_ = 0.993	*k* = −4→5
10934 measured reflections	*l* = −24→21

### Refinement

**Table:**

Refinement on *F*^2^	Primary atom site location: structure-invariant direct methods
Least-squares matrix: full	Secondary atom site location: difference Fourier map
*R*[*F*^2^ > 2σ(*F*^2^)] = 0.062	Hydrogen site location: inferred from neighbouring sites
*wR*(*F*^2^) = 0.129	H atoms treated by a mixture of independent and constrained refinement
*S* = 1.06	*w* = 1/[σ^2^(*F*_o_^2^) + (0.041*P*)^2^ + 0.7373*P*] where *P* = (*F*_o_^2^ + 2*F*_c_^2^)/3
2121 reflections	(Δ/σ)_max_ < 0.001
178 parameters	Δρ_max_ = 0.26 e Å^−^^3^
0 restraints	Δρ_min_ = −0.24 e Å^−^^3^

### Special details

**Table:**

Experimental. The crystal was placed in the cold stream of an Oxford Cryosystems Cobra open-flow nitrogen cryostat (Cosier & Glazer, 1986) operating at 100.0 (1) K.
Geometry. All s.u.'s (except the s.u. in the dihedral angle between two l.s. planes) are estimated using the full covariance matrix. The cell s.u.'s are taken into account individually in the estimation of s.u.'s in distances, angles and torsion angles; correlations between s.u.'s in cell parameters are only used when they are defined by crystal symmetry. An approximate (isotropic) treatment of cell s.u.'s is used for estimating s.u.'s involving l.s. planes.
Refinement. Refinement of F^2^ against ALL reflections. The weighted R-factor wR and goodness of fit S are based on F^2^, conventional R-factors R are based on F, with F set to zero for negative F^2^. The threshold expression of F^2^ > σ(F^2^) is used only for calculating R-factors(gt) etc. and is not relevant to the choice of reflections for refinement. R-factors based on F^2^ are statistically about twice as large as those based on F, and R- factors based on ALL data will be even larger.

### Fractional atomic coordinates and isotropic or equivalent isotropic displacement parameters (Å^2^)

**Table:**

	*x*	*y*	*z*	*U*_iso_*/*U*_eq_	Occ. (<1)
O1	0.02464 (12)	0.7567 (4)	0.37851 (8)	0.0251 (4)	
O2	0.18198 (12)	0.6548 (5)	0.43615 (8)	0.0264 (4)	
N1	0.25383 (14)	0.2963 (5)	0.33114 (10)	0.0204 (5)	
N2	0.10876 (15)	0.4334 (6)	0.25459 (12)	0.0243 (5)	
N3	0.20743 (19)	0.1167 (7)	0.14153 (12)	0.0328 (6)	
C1	0.20422 (17)	0.2863 (6)	0.26523 (12)	0.0201 (5)	
C2	0.25526 (17)	0.1188 (6)	0.20981 (12)	0.0221 (6)	
C3	0.35320 (18)	−0.0256 (7)	0.22796 (13)	0.0243 (6)	
C4	0.40151 (18)	−0.0059 (7)	0.29740 (13)	0.0248 (6)	
C5	0.35068 (18)	0.1562 (7)	0.34840 (13)	0.0241 (6)	
C6	0.08824 (17)	0.7727 (6)	0.43321 (12)	0.0207 (5)	
C7	0.05630 (18)	0.9358 (7)	0.50147 (12)	0.0207 (5)	
O1WA	0.3424 (4)	0.840 (3)	0.5375 (3)	0.082 (3)	0.670 (17)
H1WA	0.3803	0.6625	0.5341	0.123*	0.670 (17)
H2WA	0.2898	0.8222	0.5066	0.123*	0.670 (17)
O1WB	0.3443 (6)	0.583 (4)	0.5327 (3)	0.039 (4)	0.330 (17)
H1WB	0.3312	0.7954	0.5365	0.058*	0.330 (17)
H2WB	0.3173	0.5182	0.4919	0.058*	0.330 (17)
O2W	0.4593 (3)	0.248 (2)	0.5467 (3)	0.110 (3)	0.50
H1W2	0.5119	0.3543	0.5322	0.165*	0.50
H2W2	0.4462	0.0794	0.5187	0.165*	0.50
H4A	0.4676 (19)	−0.100 (7)	0.3090 (12)	0.027 (7)*	
H5A	0.3781 (17)	0.185 (6)	0.3960 (13)	0.022 (6)*	
H3A	0.3854 (18)	−0.140 (7)	0.1902 (13)	0.030 (7)*	
H7B	0.0722 (17)	0.759 (6)	0.5381 (12)	0.020 (6)*	
H7A	0.1062 (18)	1.118 (7)	0.5145 (12)	0.026 (7)*	
H2N2	0.076 (2)	0.445 (8)	0.2102 (16)	0.047 (9)*	
H1N3	0.142 (2)	0.154 (7)	0.1337 (14)	0.037 (8)*	
H1N2	0.0799 (19)	0.537 (7)	0.2905 (14)	0.028 (7)*	
H2N3	0.239 (2)	−0.022 (8)	0.1105 (15)	0.045 (9)*	
H1N1	0.2213 (19)	0.418 (7)	0.3676 (13)	0.030 (7)*	

### Atomic displacement parameters (Å^2^)

**Table:**

	*U*^11^	*U*^22^	*U*^33^	*U*^12^	*U*^13^	*U*^23^
O1	0.0211 (8)	0.0345 (11)	0.0203 (8)	0.0028 (8)	0.0054 (7)	−0.0005 (7)
O2	0.0188 (8)	0.0361 (11)	0.0253 (9)	0.0030 (8)	0.0074 (7)	−0.0010 (8)
N1	0.0177 (10)	0.0213 (12)	0.0235 (10)	−0.0029 (9)	0.0084 (8)	−0.0007 (9)
N2	0.0203 (11)	0.0296 (13)	0.0239 (11)	0.0020 (10)	0.0066 (9)	−0.0041 (10)
N3	0.0263 (13)	0.0450 (16)	0.0277 (12)	0.0052 (12)	0.0056 (10)	−0.0071 (11)
C1	0.0172 (11)	0.0179 (13)	0.0259 (12)	−0.0052 (10)	0.0067 (9)	0.0016 (10)
C2	0.0219 (12)	0.0213 (14)	0.0242 (12)	−0.0050 (11)	0.0074 (10)	−0.0006 (10)
C3	0.0229 (12)	0.0215 (15)	0.0304 (13)	−0.0033 (11)	0.0134 (10)	−0.0025 (11)
C4	0.0153 (12)	0.0234 (14)	0.0366 (14)	−0.0026 (11)	0.0067 (10)	0.0008 (12)
C5	0.0204 (12)	0.0251 (15)	0.0269 (13)	−0.0031 (11)	0.0028 (10)	0.0024 (11)
C6	0.0219 (12)	0.0185 (14)	0.0229 (12)	−0.0022 (11)	0.0082 (10)	0.0049 (10)
C7	0.0207 (12)	0.0212 (14)	0.0204 (12)	−0.0003 (11)	0.0035 (10)	0.0027 (11)
O1WA	0.068 (3)	0.111 (8)	0.061 (3)	−0.030 (3)	−0.026 (2)	0.022 (3)
O1WB	0.031 (4)	0.064 (9)	0.020 (3)	0.009 (4)	−0.002 (2)	−0.009 (3)
O2W	0.039 (3)	0.210 (8)	0.078 (4)	−0.015 (4)	−0.016 (3)	−0.006 (4)

### Geometric parameters (Å, °)

**Table:**

O1—C6	1.253 (3)	C4—H4A	0.93 (2)
O2—C6	1.274 (3)	C5—H5A	0.94 (2)
N1—C1	1.340 (3)	C6—C7	1.517 (3)
N1—C5	1.361 (3)	C7—C7^i^	1.513 (4)
N1—H1N1	0.96 (3)	C7—H7B	0.98 (2)
N2—C1	1.342 (3)	C7—H7A	0.97 (3)
N2—H2N2	0.90 (3)	O1WA—H1WA	0.8500
N2—H1N2	0.89 (3)	O1WA—H2WA	0.8501
N3—C2	1.371 (3)	O1WA—H1WB	0.2257
N3—H1N3	0.85 (3)	O1WB—H1WA	0.5518
N3—H2N3	0.91 (3)	O1WB—H2WA	1.2381
C1—C2	1.431 (3)	O1WB—H1WB	0.8500
C2—C3	1.383 (3)	O1WB—H2WB	0.8502
C3—C4	1.395 (3)	O2W—H1W2	0.8500
C3—H3A	0.96 (3)	O2W—H2W2	0.8501
C4—C5	1.357 (3)		
			
C1—N1—C5	123.6 (2)	C4—C5—H5A	124.5 (14)
C1—N1—H1N1	118.5 (14)	N1—C5—H5A	115.7 (14)
C5—N1—H1N1	117.9 (14)	O1—C6—O2	123.6 (2)
C1—N2—H2N2	120.3 (18)	O1—C6—C7	120.8 (2)
C1—N2—H1N2	120.5 (15)	O2—C6—C7	115.61 (19)
H2N2—N2—H1N2	119 (2)	C7^i^—C7—C6	115.4 (2)
C2—N3—H1N3	120.8 (18)	C7^i^—C7—H7B	113.3 (13)
C2—N3—H2N3	114.7 (17)	C6—C7—H7B	104.0 (13)
H1N3—N3—H2N3	118 (3)	C7^i^—C7—H7A	111.3 (14)
N1—C1—N2	118.2 (2)	C6—C7—H7A	107.7 (14)
N1—C1—C2	118.5 (2)	H7B—C7—H7A	104.4 (18)
N2—C1—C2	123.2 (2)	H1WA—O1WA—H2WA	107.4
N3—C2—C3	123.1 (2)	H1WA—O1WA—H1WB	73.7
N3—C2—C1	119.4 (2)	H2WA—O1WA—H1WB	54.6
C3—C2—C1	117.5 (2)	H1WA—O1WB—H2WA	91.7
C2—C3—C4	121.5 (2)	H1WA—O1WB—H1WB	67.4
C2—C3—H3A	116.1 (14)	H2WA—O1WB—H1WB	35.9
C4—C3—H3A	122.3 (14)	H1WA—O1WB—H2WB	118.6
C5—C4—C3	119.1 (2)	H2WA—O1WB—H2WB	72.5
C5—C4—H4A	119.8 (15)	H1WB—O1WB—H2WB	107.4
C3—C4—H4A	121.1 (15)	H1W2—O2W—H2W2	107.4
C4—C5—N1	119.7 (2)		
			
C5—N1—C1—N2	180.0 (2)	C1—C2—C3—C4	−0.8 (4)
C5—N1—C1—C2	0.2 (3)	C2—C3—C4—C5	0.6 (4)
N1—C1—C2—N3	−177.7 (2)	C3—C4—C5—N1	0.0 (4)
N2—C1—C2—N3	2.6 (4)	C1—N1—C5—C4	−0.4 (4)
N1—C1—C2—C3	0.4 (3)	O1—C6—C7—C7^i^	1.6 (4)
N2—C1—C2—C3	−179.4 (2)	O2—C6—C7—C7^i^	−177.9 (3)
N3—C2—C3—C4	177.2 (2)		

Symmetry codes: (i) −*x*, −*y*+2, −*z*+1.

### Hydrogen-bond geometry (Å, °)

**Table:**

*D*—H···*A*	*D*—H	H···*A*	*D*···*A*	*D*—H···*A*
N2—H2N2···O1^ii^	0.90 (3)	2.14 (3)	2.978 (3)	155 (3)
N3—H1N3···O1^ii^	0.85 (3)	2.14 (3)	2.993 (3)	176 (3)
N3—H2N3···O1WA^iii^	0.91 (3)	2.34 (3)	3.243 (6)	172 (2)
O1WA—H2WA···O2	0.85	1.93	2.764 (5)	165
N2—H1N2···O1	0.89 (3)	2.04 (3)	2.929 (3)	175 (2)
N1—H1N1···O2	0.96 (3)	1.69 (3)	2.643 (3)	171 (2)

Symmetry codes: (ii) −*x*, *y*−1/2, −*z*+1/2; (iii) *x*, −*y*+1/2, *z*−1/2.
